# Identifying patterns of Long-COVID diagnosis pathways: a Latent Class Analysis of Welsh clinical data

**DOI:** 10.23889/ijpds.v9i5.2611

**Published:** 2024-09-18

**Authors:** Hoda Abbasizanjani, Stuart Bedston, Lucy Robinson, Matthew Curds, Ashley Akbari

**Affiliations:** 1 Population Data Science, Swansea University; 2 Welsh Government

## Objectives

Long-COVID (LC) diagnosis remains challenging due to its variable, multisystem, episodic symptoms, and lack of uniformity in the critical time points. We aim to classify diagnosis pathways and associated demographic and health determinants for clinically diagnosed LC patients in Wales.

## Approach

We created a cohort of LC-diagnosed adults in Wales from 2020 to 2023 using population-scale data within the SAIL Databank. To classify diagnosis pathways, we extracted a set of indicators suggestive of the general diagnosis process within 6-months before the diagnosis, including LC symptoms, clinical investigations, and management. We conducted Latent Class Analysis (LCA) to examine how these indicators cluster, adjusting the best-fit model for various covariates such as COVID-19 infection history and sociodemographic characteristics.

## Results

Of the 7,973 identified LC patients, 59% had clinical investigations, 59% had a clinical management record, and 39% had at least one related symptom. Using LCA, we identified 7 distinct patterns of diagnosis pathways, and how background characteristics associated with each.

## Conclusion

Despite existing guidelines, LC diagnosis is complex due to LC’s diverse manifestations and the absence of exclusive symptoms/tests. Late or missed LC diagnosis can have a detrimental effect at individual, community, and national levels. Previous UK studies have indicated low recording rates of LC compared with self-reported surveys. Understanding current diagnosis pathways can shed light on this discrepancy and identify potential confounding factors that delay/impact LC diagnosis. This insight is crucial for standardising diagnosis pathways, enabling earlier and more accurate detection of LC and thereby mitigating its impact.

